# Leptin Levels in Serum or Semen and Its Association with Male Infertility: A Meta-Analysis with 1138 Cases

**DOI:** 10.1155/2022/9462683

**Published:** 2022-09-29

**Authors:** Yi Mo, Fangfang Liang, Arshad Mehmood, Xiangli Niu, Ying Xie, Suleman Shah, Zhong Lin, Yan Sun, Daxian Tan, Yafen Guo, Hesheng Jiang

**Affiliations:** ^1^The Reproductive Hospital of Guangxi Zhuang Autonomous Region, Nanning 530029, China; ^2^Guangxi University of Chinese Medicine, Nanning 530200, China; ^3^College of Animal Science and Technology, Guangxi University, Nanning 530001, China; ^4^Department of Neurology, The Second Hospital of Hebei Medical University, Shijiazhuang 050000, Hebei, China; ^5^Department of Genetics, Hebei Medical University, Hebei Key Lab of Laboratory Animal, Shijiazhuang 050017, Hebei, China

## Abstract

**Background:**

Leptin has an association with male infertility. However, only sporadic studies inconsistently reported the results. *Aim and Objective.* In this study, we aimed to perform a meta-analysis to investigate the relationship between leptin and male infertility.

**Methods:**

This study was performed based on published articles related to leptin and infertile males. PubMed, Web of Science, Google Scholar, Ovid + Cochrane Central Register of Controlled Trials, Wiley Online Library, Chinese CNKI, Chinese Chong Qing VIP, Chinese Wan Fang, and China Biology Medicine databases were searched to identify all relevant studies. All eligible works of literature were analyzed by the “meta” or “metan” command in STATA version 12.0 software. The standardized mean difference (SMD) of leptin concentration in serum or semen and 95% confidence intervals (CIs) were estimated for all studies. The heterogeneity was described with I^2^. The sources of heterogeneity were explored via metaregression, and stratified analyses, sensitivity analyses, and publication bias were performed.

**Results:**

Nineteen studies were included in the current meta-analysis, involving 1138 cases of infertile men and 756 controls. The SMD of leptin concentration in serum was 2.002 (95% CI: 1.086, 2.918), Z-test (*z*) *z* = 4.29; *p* < 0.001, and I^2^ was 97.3%, *p* < 0.001. The SMD of leptin concentration in semen was 3.274 (95% CI: 2.137, 4.411), z = 5.64; *p* < 0.001, and I^2^ was 98.2%, *p* < 0.001. Notably, serum follicle-stimulating hormone (FSH) was slightly higher in infertile men (SMD = 3.695, *z* = 2.33, *p* = 0.020, I^2^ = 98.8%, *p* < 0.001). Other hormones, such as luteinizing hormone (LH) and testosterone, were also slightly higher, but the results were not statistically significant. In addition, sperm count (SMD = −4.533, 95% CI: −6.565, −2.501) and sperm motility (SMD = −7.894, 95% CI: −10.616, −5.172) inversely correlated with leptin levels in infertile males. Sperm abnormal forms did not show a statistically significant SMD of −0.076 (95% CI: −3.410, 3.258).

**Conclusion:**

Leptin plays a potential role in association with male infertility. This study may effectively reveal the relationship between leptin together with other hormones and its association with male infertility. These results may also provide opinions on precautionary measures.

## 1. Introduction

The proportion of infertile couples of childbearing age has been increasing every year. Infertility affects roughly 15% of couples of reproductive age, and the malefactor accounts for 20% to 50% of all cases [1, 2]. Declines in sperm concentration and sperm motility have been reported in recent years [3]. Increasing rates of obesity and metabolic syndrome have been linked to subfertility or infertility in men. Notably, considering progress in research on reproductive physiology, studies have shown that leptin is inextricably linked with reproduction, especially in male infertility [4–6]. Leptin, an adipocyte-derived protein hormone, supports the body in maintaining homeostasis [7]. Together with other adipokines, leptin is responsible for the inflammatory state found in obesity and with both direct and indirect effects that lead to type 2 diabetes, metabolic syndrome, and cardiovascular disease [8]. Numerous studies have reported that leptin plays a vital role in male fertility dysfunction. However, there is no meta-analysis about leptin and male fertility; thus, the relationship between leptin and male fertility has been elusive.

Considering the sporadic prior research, we used the updated meta-analysis to investigate the relationship between leptin concentration and the association of male fertility dysfunction, and we aimed to provide new clues for the etiology of male infertility.

## 2. Materials and Methods

### 2.1. Search Strategy

A systematic search of PubMed, Embase, Web of Science, Google Scholar, Ovid + Cochrane Central Register of Controlled Trials, Wiley Online Library, China National Knowledge Infrastructure (CNKI), as well as the Chinese Chongqing VIP, Chinese Wan Fang, and China Biology Medicine (CBM) databases was conducted to distinguish all the applicable studies published on or before June 2, 2018, with the following search strategy: (“leptin” OR “LEP” OR “obese protein” OR “obese gene product” OR “adipokine” OR “adipocytokine”) AND (“male infertile” OR “male subfertile”).

### 2.2. Study Selection and Inclusion Criteria

In this meta-analysis, published studies were eligible for inclusion if they confirmed all of the following criteria: (1) the articles should illuminate leptin with the risk of male infertility and should contain sufficient information; (2) studies were excluded if they were case reports, summaries, minutes of meetings, unpublished articles, letters, and repeated documents; (3) study design using a case-control study or cohort study; (4) studies published in both Chinese and English were included. As the last criterion, if there were more than two articles from the same team, the one with a larger sample size was included. Screening of documents was operated separately by two researchers, eliminating differences by discussing or consulting a third party.

### 2.3. Data Extraction

The following data were extracted: name of the first author, year of publication, country, body mass index (BMI), sample size (n), leptin test method, materials for the test, number of cases and controls, mean age, other hormones, and semen parameters.

### 2.4. Statistical Analysis

The whole meta-analysis and pictures were performed on STATA software, version 12.0. The aim was to clarify the relationship between leptin in serum or semen and the risk of male infertility. Among the 19 case-control studies, the SMD and 95% CIs were computed. The random-effect model was selected to evaluate statistical heterogeneity between different trials. Subgroup analysis was applied to analyze the relationships among 19 case-control studies and to identify the sources of heterogeneity. A quantified description of heterogeneity was performed based on *I*^2^ and a *p*value of more than 0.05. *I*^2^ lower than 50% was defined as low heterogeneity. Funnel plots were used to assess publication bias. *p* < 0.05 was significant for the whole article.

## 3. Results

### 3.1. Eligible Studies

**Figure 1:**
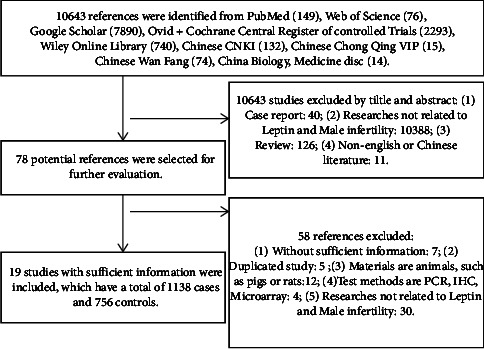
The flowchart of the search process with the number of screened, excluded, and included publications.

The procedure of the meta-analysis is shown in Figure 1. A total of 10643 related publications were identified based on the primary search strategy. With the titles and abstracts being glanced over, 78 potential references were studied for further evaluation as eligible in accordance with the earlier stated criteria. By reviewing the full text rigorously, 58 articles were eliminated, including 7 references without sufficient information, 5 reduplicated studies, 12 studies conducted in nonhuman tissues, including materials from animals, such as pigs or rats, 4 studies which detected serum leptin concentrations with polymerase chain reaction (PCR), immunohistochemistry (IHC), and microarray, and 30 references which were not related to leptin and male infertility. Notably, 19 references were studied with sufficient information in this meta-analysis, of which 12 were in English and 7 in Chinese, and a total of 1138 cases and 756 controls were included in 19 studies (Tables 1 and 2).

### 3.2. Characteristics of the Study

Among all 19 studies, 10 were performed in mainland China. Among all studies, materials were either serum or semen. Serum or seminal leptin was detected through an enzyme-linked immunosorbent assay (ELISA) based technique in 8 studies, while 11 studies used radioimmunoassay (RIA). In addition, the male infertility types and the corresponding number of studies were, respectively, obstructive azoospermia (OBST-NON-OBSTAZOOs), nonobstructive azoospermia (NON-OBSTAZOOs) [3], oligozoospermia (OZS) [2], asthenozoospermic [4], teratozoospermia [3], and other rare types [5]. If a study provided more than one type of male infertility without overall pooled results, we would choose the type with the larger sample size. Subsequently, 7 studies provided data about other hormones such as FSH, LH, and testosterone (*T*). The detailed information is presented in Table 1.

### 3.3. Serum or Semen Leptin Concentration in Infertile Males

**Figure 2:**
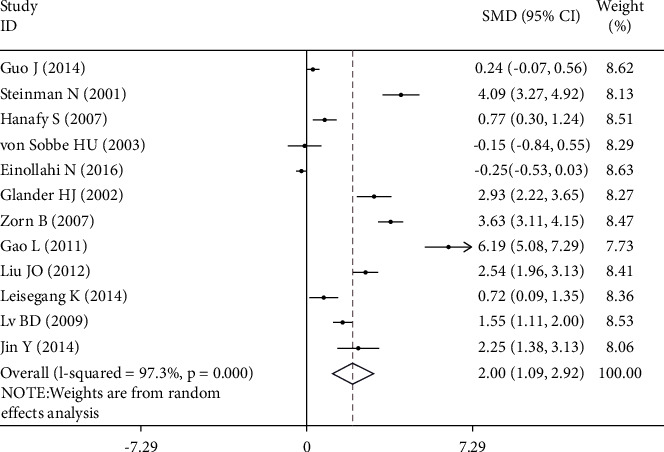
The SMD of leptin concentration in serum and male infertility risk. SMD: standardized mean difference. Note: weights are from the random-effects analysis.

**Figure 3:**
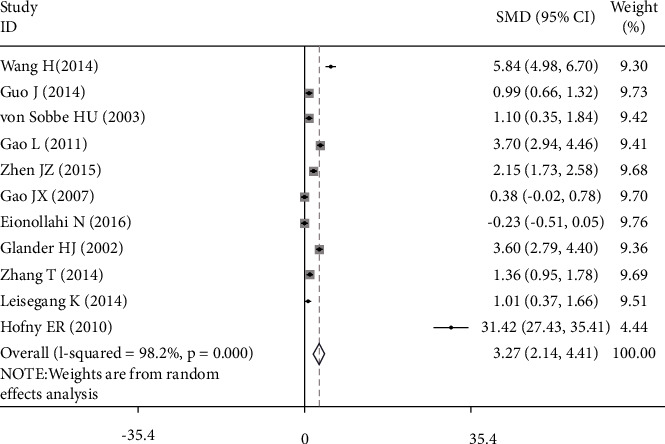
The SMD of leptin concentration in seminal and male infertility risk. SMD: standardized mean difference. Note: weights are from the random-effects analysis.

The serum or semen leptin concentration was depicted by decimals on forest plots. The SMD, with its 95% CI, was estimated from 1138 cases and 756 controls based on a random-effect model (Figures 2 and 3). The SMD of leptin concentration in serum was 2.002 (95% CI: 1.086, 2.918, *z* = 4.29; *p* < 0.001), and obvious heterogeneity was found among various articles (*I*^2^ was 97.3%, *p* < 0.001) (Figure 2). The SMD of leptin concentration in semen was 3.274 (95% CI: 2.137, 4.411) (Figure 3) using the random-effect model, which was statistically significant (z = 5.64; *p* < 0.001), and obvious heterogeneity was found among various articles (I2 = 98.2%, *p* < 0.001). The result denoted that serum or seminal leptin in infertile males was higher than that in normal fertile men. However, the existence of heterogeneity could not be ignored. Stratified analysis, metaregression, and sensitivity analysis were used to explore the source of heterogeneity.

### 3.4. Serum or Semen Leptin Concentration in Subgroups

**Figure 4:**
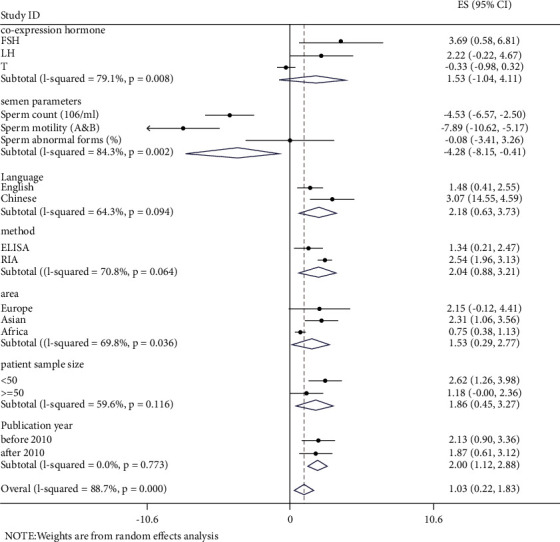
The estimates of SMD are based on 12 eligible studies providing serum leptin concentration with their 95% CIs. Note: FSH: follicle-stimulating hormone; LH: luteinizing hormone; T: testosterone; ELISA: enzyme-linked immunosorbent assay; RIA: radioimmunoassay.

**Figure 5:**
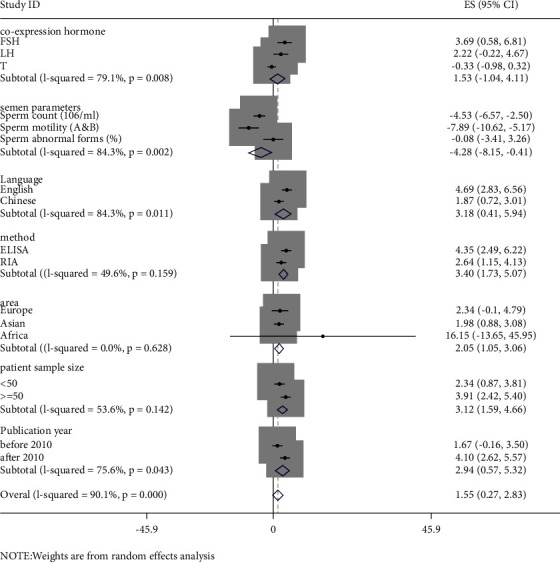
The estimates of SMD are based on 12 eligible studies providing seminal leptin concentration with their 95% CIs. Note: FSH: follicle-stimulating hormone; LH: luteinizing hormone; T: testosterone; ELISA: enzyme-linked immunosorbent assay; RIA: radioimmunoassay.

The studies can be classified by their materials, test method, area, language, patient sample size, and the publication year (Table 1, Figures 4, and 5). The serum leptin concentration was higher in cases using RIA than those using ELISA methods (SMD = 2.541 versus SMD = 1.341), but heterogeneity cannot be ignored (I2 = 97.3%, *p* < 0.001). However, the seminal leptin concentration was higher in cases using ELISA than those using RIA methods (SMD = 4.353 versus SMD = 2.637), but heterogeneity was still obvious (*I*^2^ = 98.2%, *p* < 0.001). Simultaneously, there was no statistical significance between Europe and Africa regarding the SMD of seminal leptin concentration. The SMD of serum leptin in cases in Europe (SMD = 2.146) was similar to cases in Asia (SMD = 2.309), and the SMD of serum leptin in both continents were higher than that in Africa (SMD = 0.751). However, there was no statistical significance in cases in Europe (Table 1, Figures 4 and 5).

The SMD of serum leptin in cases in China (3.069) was higher than that in non-Chinese regions (1.479). The SMD of serum leptin in large samples (>50 cases) was 1.178, and in small samples (<50 cases), it was 2.623. However, the SMD of seminal leptin in cases in China (1.865) was lower than that in non-Chinese regions (4.694). The SMD of seminal leptin in large samples (>50 cases) was 3.910, and in small samples (<50 cases), it was 2.342. There was no remarkable difference between works of literature published before or after 2010, with an SMD of serum leptin of 2.128 and 1.869, respectively. In contrast, the SMD of serum leptin in works of literature published before 2010 was much lower than that after 2010 (1.667 versus 4.096).

### 3.5. Serum or Semen Leptin Concentration and Other Coexpression Hormones

Eight studies provided data on other coexpression hormones (including FSH, LH, and T). The SMD of FSH, LH, and T was 3.695, 2.224, and −0.331, respectively, whereas the results of LH and T were statistically insignificant. These three coexpressions of hormones and unignorable heterogeneity were studied. Other studies that provided coexpression hormones, such as prolactin (PRL) and estrogen, had insufficient information; thus, data were not analyzed in this study (Figures 4 and 5).

### 3.6. Serum or Semen Leptin Concentration and Its Relationship with Semen Parameters

Seven studies provided concrete information on the semen parameters of different types of infertile and normal males. It turned out that the sperm count and sperm motility were obviously weaker in infertile males than those in normal males. The SMD of the sperm count (10^6^/mL) was −4.533 (95% CI: −6.565, −2.501), *z* = 4.37; *p* < 0.001 (*I*^2^ = 98.1%, *p* < 0.001), and sperm motility (A and B) was −7.894(95% CI: −10.616, −5.172), *z* = 5.68; *p* < 0.001 (*I*^2^ = 99.0%, *p* < 0.001), whereas, sperm morphology abnormal forms (%) had insignificant outcomes results, SMD = −0.076 (95% CI: −3.410, 3.258), *z* = 0.04; *p*=0.964 (Figures 4 and 5).

### 3.7. Sensitivity Analysis and Publication Bias

**Figure 6:**
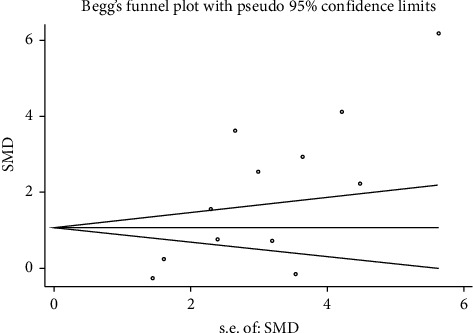
Publication bias via Begg's test based on works of the literature providing the serum leptin concentration (continuity-corrected: *z* = 2.13, *p*=0.034).

**Figure 7:**
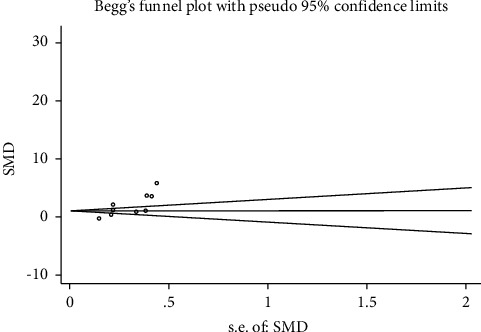
Publication bias via Begg's test based on works of the literature providing the seminal leptin concentration (continuity-corrected: *z* = 3.27, *p*=0.001).

Sensitivity analysis implied that each study made almost no significant difference in the pooled effect estimate except for a study by Hofny et al. [9]. Metaregression was performed, and no sources of heterogeneity were observed in any of the factors, such as test method, area, language, patient sample size, and published year. Begg's test was conducted, and there was obvious publication bias with the serum and seminal leptin concentrations (Figures 6 and 7, respectively).

## 4. Discussion

The World Health Organization (WHO) has pointed out that couples of normal childbearing age who have regular sex without using contraceptive measures for more than 12 months resulting in conception failure can be diagnosed as infertile. According to the survey by Esteves SC, about 10%–15% of noncontraceptive childbearing age couples in the world are unable to give birth because of various factors, of which 50% are malefactors [1]. Male infertility is clinically divided into sexual dysfunction and sexual function. The latter can be further divided into azoospermia, oligozoospermia, asthenozoospermia, spermatogenesis, and sperm sterility based on semen analysis. The main causes of male infertility are decreased semen quality, impaired sperm production and maturation, and blockage of sperm transport channels. Human fertility is linked to sperm quality and hormonal regulation of spermatogenesis [10–12].

It has been suggested that leptin is a metabolic signal to the reproductive system. Adipokines attract substantial interest in the context of male infertility since they play a key role in regulating energy homeostasis, and aberrant secretion of adipokines is associated with the etiology of several reproductive abnormalities [13–17].

In light of progress in research on reproductive physiology, studies have shown that leptin is inextricably linked with reproduction, especially in male infertility [18, 19]. Leptin, the product of the obese (OB) gene, is a cytokine synthesized and secreted mainly by adipocytes [20]. It inhibits food intake and regulates energy metabolism by inducing anorexigenic factors and suppressing orexigenic neuropeptides [21]. The published studies have suggested that food consumption is associated with a transient increase in serum leptin levels, whereas fasting reduces OB gene expression [20]. However, serum levels of leptin can be disturbed by other factors, such as various proinflammatory cytokines, including tumor necrosis factor-alpha (TNF-a), interleukin-1 (IL-1), and interleukin-6 (IL-6) [22]. It is expressed mainly on spermatocytes in seminiferous tubules, and its expression increases with spermatogenic dysfunction [23]. Leptin receptors are present in testicular tissue [24]. The mechanism of leptin action seems to be inhibition of T secretion, as revealed in *in vitro* studies in the adult rat testis [25]. The findings regarding the secretion of leptin from human spermatozoa accompanied by the presence of leptin receptors on human spermatozoa and in seminal plasma may open a novel field of study in male infertility [26, 27].

In our study, serum leptin levels were measured in normal and infertile male subjects with different categories of infertile patients. Significantly elevated leptin concentrations were observed in infertile male patients. However, we found nonignorable heterogeneity (*I*^2^ = 96.2%) between studies in meta-analysis. Although heterogeneity can be considered by using the random-effect model, it would increase the probability of type-I error. The following factors may have contributed to the significant heterogeneity: (1) References from 2001 to 2016 are included. Three versions of the WHO laboratory manual for examination and processing were used, and criteria for human semen parameter standards and related definitions are inconsistent, such as, in the fifth version since 2011, “normozoospermic men” were defined as the semen parameter as ≥15×106 million of sperms/ml, ≥40% total motility, ≥32% progressive motility, and ≥4% normal forms, while in the fourth version since 2006, ≥20×106 million of sperms/ml, ≥75% total motility, ≥50% progressive motility, and ≥15% normal forms.. (2) The inclusion criteria of the case group are also inconsistent. In some reference experiments, only nonobstructive azoospermia patients were included, while in the other reference experiments, not only the nonobstructive patients but also the oligoasthenoteratozoospermic patients were included. (3) The demographic characteristics and genetic backgrounds were different in Europe, Asia, and African populations. (4) The studied infertile males showed different types of pathogenesis. (5) The literature published in different years were included in the studies. (6) There were different BMIs of patients in each primary study because obesity infertile and obese healthy males can lead to slightly increased leptin concentration. (7) Different measuring methods were used in the primary studies. (8) The included studies were of differing qualities. To identify the sources of heterogeneity, first, we carried out a sensitivity analysis that indicated the stability of the meta-analysis. Thus, we found that each study made almost no significant difference in the pooled effect estimate except for a study by Hofny et al. [9]. Second, we also conducted a multivariate metaregression analysis to assess the possible confounding factors further, but the sources of heterogeneity were not observed. The confounding factors do not substantially affect heterogeneity, such as test method, area, language, patient sample size, and published year. Therefore, we finally conducted subgroup analyses of different specific effects. The serum or seminal leptin levels slightly differed between the infertile male patients and fertile ones in subgroup analyses. According to the Newcastle–Ottawa scale (NOS), each study in our meta-analysis scored 6 points or more. However, publication bias was found by performing Begg's and Egger's tests [28, 29].

It is the first meta-analysis of the relationship between serum or seminal leptin concentration and the risk for male infertility. Nineteen studies were included in the current meta-analysis, involving 1138 cases of infertile men and 756 controls. We found that serum or seminal leptin was higher in infertile males than that in fertile ones. The SMD of leptin concentration in serum was 2.002 (95% CI: 1.086, 2.918), *z* = 4.29; *p* < 0.001, while *I*^2^ was 97.3%, *p* < 0.001. The SMD of leptin concentration in semen was 3.274 (95% CI: 2.137, 4.411), *z* = 5.64; *p* < 0.001 (*I*^2^ = 98.2%, *p* < 0.001), and the leptin concentration in semen especially in fertile males was over three-fold. Simultaneously, FSH also increased in fertile males with statistical significance. However, the mechanism of the contribution of leptin to male spermatogenic function is still elusive. In this study, both seminal and serum leptin were increased and correlated with male infertility. Some potential effects of leptin on the male gonads have been reported. It is suggested that leptin may regulate the secretion of inhibin B or may even be related to regulating the function of Sertoli cells. Leptin may affect both Sertoli and Leydig cells. These could be some of the ways through which it affects spermatogenesis [30].

Numerous studies have been carried out to determine the association of leptin in the regulation of the reproductive axis. Human fertility is associated with sperm quality and hormonal regulation of spermatogenesis. Significantly higher serum concentrations of leptin, FSH, and LH were found in infertile males. In contrast, T concentrations were found to be lower in infertile males. Inverse correlation can be explained by the paracrine inhibitory effect of leptin, FSH, and LH on T production by Leydig cells [12].

## 5. Limitations

Several limitations exist in this study and deserve discussion. First, the number of primarily included studies was very small. Second, the pathogenesis types of male infertility were complicated. The heterogeneity we found in the included studies may be different depending on the in-study methods, case-control selection, and statistical analysis tools. By using a random-effect model for more estimates, we addressed these issues. Studies that were restricted only to test methods and leptin detection in human tissues were included. Some studies based on animals were excluded. Notably, we could not exclude biases even though it had evident publication bias which was found in Begg's test. This study indicated that serum or seminal leptin was statistically associated with male infertility.

## 6. Future Perspectives

In the present study, leptin plays a potential role in association with male infertility. Overall, higher leptin concentration in infertile males was associated with a slightly higher sex hormone concentration, such as FSH, LH, and T, which were inversely correlated with a lower risk of the sperm count and sperm motility. This result may provide an opinion on the diagnosis of male infertility etiology and provide a potential precautionary measure for infertile males, especially obese man, who always has higher leptin concentration in serum.

## 7. Conclusion

This study suggests that serum and tissue leptin are associated with male infertility. However, the role of leptin in the regulation of the reproductive axis is still elusive. Further study is needed to explore the mechanism of how leptin is potentially associated with male infertility.

## Figures and Tables

**Table 1:** The characteristics of 12 eligible studies providing serum leptin concentration.

Year	Country	Control						Case						Language	Method
		Total (*n*)	Leptin (*u*g/L)	Age (*y*)	BMI	Sperm concentration (10^6^/mL)	Sperm progressive motility (%)	Total (*n*)	Leptin (*u*g/L)	Age (*y*)	BMI	Sperm concentration (10^6^/mL)	Sperm progressive motility (%)		
2014	China	77	9.22 ± 1.15	31.1 ± 2.7	24.32 ± 2.14	41.84 ± 11.39	48.73 ± 7.27	79	9.48 ± 0.98	30.9 ± 2.7	24.15 ± 2.13	33.47 ± 11.08	20.42 ± 5.78	English	ELISA
2001	Israel	35	5 ± 0.4	31.6 ± 1.4	<25	≥20	≥50	36	7.6 ± 0.8	36.6 ± 1.3	<25	≤8.5 ± 6.7	≤16 ± 5.5	English	RIA
2007	Egypt	30	6.88 ± 8.65	30.9 ± 8.8	27.3 ± 6.4	71.35 ± 35.7	N/A	50	16.3 ± 13.98	30.1 ± 7.3	28.15 ± 4.4	8.09 ± 4.8	N/A	English	RIA
2003	Germany	21	5.62 ± 3.72	33 ± 4	23 ± 3	88.31 ± 41.81	≥50	13	5.12 ± 2.93	33 ± 4	23 ± 3	0	0	English	RIA
2016	Iran	95	7.69 ± 6.9	34.12 ± 5.3	24.15 ± 3.8	27.6 ± 16.5	22.98 ± 18.9	98	6.1 ± 5.8	34.14 ± 6.2	24.17 ± 4.42	9.45 ± 8	22.51 ± 11.64	English	ELISA
2002	Germany	28	2.9 ± 0.6	N/A	25.45 ± 2.45	86.3 ± 15.2	31.9 ± 2.6	36	4.5 ± 0.5	N/A	25.60 ± 2.46	23.6 ± 4.1	18.1 ± 1.9	English	RIA
2007	Slovenia	85	8.2 ± 0.6	34.6 ± 0.4	25.54 ± 0.31	≥20	≥50	68	12.4 ± 1.6	34.6 ± 0.4	26.22 ± 0.47	0	0	English	ELISA
2011	China	30	5.61 ± 0.43	29.8 ± 0.57	21.83 ± 1.36	≥20	≥32	45	13.29 ± 1.56	29.56 ± 0.77	23.45 ± 0.57	0	0	Chinese	RIA
2012	China	40	7.88 ± 2.63	30.42 ± 6.22	N/A	24.58 ± 4.13	43.97 ± 4.89	42	16.32 ± 3.86	29.14 ± 6.01	N/A	18.56 ± 3.24	27.90 ± 5.03	Chinese	RIA
2014	South Africa	19	4.1 ± 2.4	35.1 ± 5.9	25.5 ± 2.4	35.3 ± 16.7	33.8 ± 16.2	23	8.8 ± 8.5	37.9 ± 7.3	35.8 ± 4.3	23.7 ± 13.6	24.5 ± 19.1	English	ELISA
2009	China	48	3.86 ± 1.56	32 ± 5.24	18.56 ± 2.32	60.38 ± 8.62	75.80 ± 4.38	52	16.82 ± 11.46	32 ± 5.24	30.21 ± 2.9	22.90 ± 2.15	28.12 ± 4.14	Chinese	ELISA
2014	China	10	4.22 ± 0.76	31.1 ± 4.38	22.74 ± 1.43	40.49 ± 12.02	31.28 ± 9.41	30	7.63 ± 1.68	29.6 ± 3.21	26.79 ± 1.28	15.54 ± 3.45	12.54 ± 8.36	Chinese	ELISA

ELISA: enzyme-linked immunosorbent assay; RIA: radioimmunoassay; N/A: negative.

**Table 2:** The characteristics of 12 eligible studies providing seminal leptin concentration.

Year	Country	Control						Case						Language	Method
		Total (*n*)	Leptin (*u*g/L)	Age (*y*)	BMI	Sperm concentration (10^6^/mL)	Sperm progressive motility (%)	Total (*n*)	Leptin (*u*g/L)	Age (*y*)	BMI	Sperm concentration (10^6^/mL)	Sperm progressive motility (%)		
2014	China	40	1.69 ± 0.11	28.83 ± 5.35	22.41 ± 1.28	21.86 ± 2.57	82.62 ± 4.23	74	3.2 ± 0.31	29.76 ± 13.18	22.31 ± 0.85	17.98 ± 3.60	37.31 ± 8.08	English	RIA
2014	China	77	3.75 ± 0.97	31.1 ± 2.7	24.32 ± 2.14	41.84 ± 11.39	48.73 ± 7.27	79	4.72 ± 0.99	30.9 ± 2.7	24.15 ± 2.13	33.47 ± 11.08	20.42 ± 5.78	English	ELISA
2003	Germany	21	0.21 ± 0.21	33 ± 4	23 ± 3	88.31 ± 41.81	≥50	13	0.54 ± 0.41	33 ± 4	23 ± 3	0	0	English	RIA
2011	China	30	1.69 ± 0.2	29.8 ± 0.57	21.83 ± 1.36	≥15	≥32	45	4.8 ± 1.07	29.56 ± 0.77	23.45 ± 0.57	0	0	Chinese	RIA
2015	China	62	0.96 ± 0.42	32.8 ± 6.2	N/A	≥15	≥32	76	2.39 ± 0.81	33.4 ± 5.9	N/A	<15	N/A	Chinese	ELISA
2007	China	30	5.29 ± 2.11	30.3 ± 4.2	<25	N/A	N/A	126	6.3 ± 2.79	30.4 ± 4.0	<25	N/A	N/A	Chinese	RIA
2016	Iran	95	0.65 ± 0.38	34.12 ± 5.3	24.15 ± 3.8	27.6 ± 16.5	22.98 ± 18.9	98	0.56 ± 0.4	34.14 ± 6.2	24.17 ± 4.42	9.45 ± 8	22.51 ± 11.64	English	ELISA
2002	Germany	28	1.5 ± 0.2	N/A	25.45 ± 2.45	86.3 ± 15.2	31.9 ± 2.6	36	3.19 ± 0.6	N/A	25.60 ± 2.46	23.6 ± 4.1	18.1 ± 1.9	English	RIA
2014	China	40	1.58 ± 0.23	27.69 ± 6.53	21.91 ± 0.76	26.29 ± 9.40	54.85 ± 9.76	80	2.81 ± 1.09	28.16 ± 7.62	22.92 ± 0.42	18.16 ± 8.87	25.23 ± 12.72	Chinese	RIA
2014	South Africa	19	5.6 ± 3.8	35.1 ± 5.9	25.5 ± 2.4	35.3 ± 16.7	33.8 ± 16.2	23	12.9 ± 9.1	37.9 ± 7.3	35.8 ± 4.3	23.7 ± 13.6	24.5 ± 19.1	English	ELISA
2010	Egypt	42	7.93 ± 0.05	29.79 ± 1.1	31.21 ± 0.39	51.57 ± 6.70	57.50 ± 1.41	80	12.36 ± 0.17	29.35 ± 0.9	33.49 ± 0.43	12.97 ± 1.55	13.25 ± 0.79	English	ELISA

ELISA: enzyme-linked immunosorbent assay; RIA: radioimmunoassay; N/A: negative.

## Data Availability

The data supporting the meta-analysis are included in the article.
